# Porcine Cysticercosis in Southeast Uganda: Seroprevalence in Kamuli and Kaliro Districts

**DOI:** 10.1155/2009/375493

**Published:** 2009-06-28

**Authors:** C. Waiswa, E. M. Fèvre, Z. Nsadha, C. S. Sikasunge, A. L. Willingham

**Affiliations:** ^1^Faculty of Veterinary Medicine, Makerere University, P.O. Box 7062, Kampala, Uganda; ^2^Centre for Infectious Diseases, School of Biological Sciences, University of Edinburgh, Ashworth Laboratories, Kings Buildings, West Mains Road, Edinburgh EH9 3JT, UK; ^3^School of Veterinary Medicine, University of Zambia, P.O. Box 32379, Lusaka, Zambia; ^4^WHO/FAO Collaborating Centre for Research and Training for Emerging and Other Parasitic Zoonoses, Parasitology, Health and Development Section, Department of Veterinary Disease Biology, Faculty of Life Sciences, University of Copenhagen, Dyrelægevej 100, 1870 Frederiksberg C, Denmark

## Abstract

The recent recognition of neurocysticercosis as a major cause of epilepsy in Uganda and changes in pig demography have lead to a need to better understand the basic epidemiology of *Taenia solium* infections in pigs and humans. Human exposure is a function of the size of the animal reservoir of this zoonosis. This is the first field survey for porcine cysticercosis to investigate the prevalence of antigen-positive pigs across an entire rural district of south-east Uganda. In our field surveys, 8.6% of 480 pigs screened were seropositive for the parasite by B158/B60 Ag-ELISA. In addition, of the 528 homesteads surveyed 138 (26%) did not have pit latrines indicating a high probability of pigs having access to human faeces and thus *T. solium* eggs. This study thus indicates the need for better data on this neglected zoonotic disease in Uganda, with a particular emphasis on the risk factors for infection in both pigs and humans. In this regard, further surveys of pigs, seroprevalence surveys in humans and an understanding of cysticercosis-related epilepsy are required, together with risk-factor studies for human and porcine infections.

## 1. Introduction

Pig-keeping and pork consumption in Eastern and Southern Africa (ESA) are rising rapidly [1] as demand for pork increases and rural and peri-urban families discover pig farming to be profitable and cost effective [2]. In Uganda, the establishment of piggeries and increased pig production by rural farmers is encouraged by government and forms a part of central government agricultural planning [3]. In some instances, local governments are supplying piglets to rural families in order to promote an alternative source of income. Pigs are considered low-input livestock which, as accomplished scavengers, can grow to market size on minimal feed inputs from the farmer. Humans serve as the definitive host of the zoonotic helminth parasite *Taenia solium*, harbouring the adult tapeworm (infection termed taeniosis), which is acquired through the consumption of under-cooked pork (the larval cysticerci in the consumed pork develop into adult tapeworms in the human gut). Pigs acquire larval *T. solium * infection (infection termed cysticercosis) by ingestion of *T. solium * eggs passed in human faeces (i.e., coprophagy). Humans may also be infected with cysticercosis from ingestion of *T. solium * eggs through faecal-oral contamination. If cysts form in human brain tissue, a neurological disorder known as neurocysticercosis results; this commonly manifests as epileptic seizures [4]. Cysticercosis is acquired in settings with poor household and/or personal hygiene and thus can be acquired by both pork eaters and nonpork eaters; *T. solium * is emerging as a serious agricultural and public health problem in the ESA region [5]. 

In eastern Africa, the disease has been reported in Tanzania [6], Kenya [2], Uganda [7], Burundi [8, 9] and Rwanda [10], though it is known to be substantially under-reported in both humans and pigs. Epidemiological field research is required for improving our understanding of both the prevalence and risk factors of this parasitic disease, while also investigating the transmission dynamics [11, 12]. The aim of the present work is to determine the prevalence of cysticercosis among domestic pigs by B158/B60 Ag-ELISA in the districts of Kamuli and Kaliro, south-east Uganda, to provide baseline information on the level of exposure in this region. Anecdotally, Kamuli is known to be a source of pig meat for the burgeoning demand of urban Kampala, the capital city, and consumption of pork meat amongst the local population is also extensive. There have been no recent epidemiological studies on human taeniasis, human cysticercosis or porcine cysticercosis in Uganda; assessing the infection rate in the pig population has been shown to be a good indicator of *T. solium * risk to humans [13].

## 2. Materials and Methods

### 2.1. Study Area

**Figure 1:**
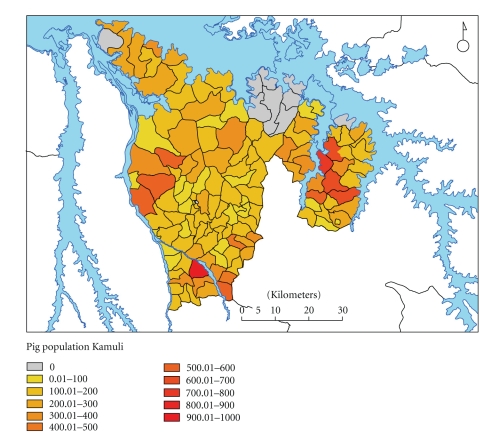
Pig population of the study area (Kamuli and Kaliro districts). Data from the District Veterinary Office, Kamuli (survey undertaken in 2003-2004).

Kamuli and Kaliro districts in Uganda were chosen for the study as pig keeping is known to have become popular in these districts. Kaliro was formally part of Kamuli District (until 2005 when it became a district in its own right). The study area had an estimated pig population of 25,860, as determined from the 2003-2004 Agricultural Census conducted by the District Veterinary Office (DVO) with a spatial distribution as shown in Figure 1. The pigs can best be described as the local breeds; the percentages of the cross breeds cannot be estimated with certainty since there are no breeding records kept. The field survey reported here was carried out in October–December 2005. 

### 2.2. Cross-Sectional Sampling

Previous studies elsewhere in East Africa have found a prevalence of cysticercosis infection among pigs in some areas to be around 20% [2, 6]; no such studies have previously been published for our study area. The sample size for a cross-sectional survey to estimate prevalence was calculated based on random sampling of pigs and inflated to account for the uncertainty and multi-stage design [14]. With an expected prevalence of 20%, an accepted error of 5% and 95% confidence interval, the minimum number to sample was 246 pigs, which, doubled, was 492, rounded to 500. We aimed to sample pigs in every parish and the 500 samples were allocated to the overall total of 129 parishes (which are within 21 sub-counties). Sampling was proportionally allocated to each parish, depending on the pig population of that parish [14], so that parishes with more pigs had a higher proportion of samples. Animal health workers provided a complete list of homesteads in each parish from which homes were selected randomly and pigs to sample were also randomly chosen during the home to home visits.

### 2.3. Collection of Serum Samples, Latrine Survey and Husbandry System

Pigs were bled from the cranial vena cava and blood collected into plain vacutainer tubes and then allowed to clot at ambient temperature and later centrifuged to separate the serum which was extracted and stored at −20°C until use. The age of the pigs sampled was recorded and a total of 528 homesteads were visited for this purpose in the two districts. The pig husbandry system was assessed by direct observation and discussions with the pig owners. In each village visited during the home to home pig survey, the availability of pit latrines and information on deworming of pigs by the veterinary staff in the area was captured at every fifth homestead.

### 2.4. Analysis of Serum Samples

The B158/B60 Ag-ELISA for the detection of circulating antigens of *T. solium * cysticerci was conducted as described by Brandt et al. [15], and modified by Dorny et al. [16]. The test was carried out at the School of Veterinary Medicine, University of Zambia, Lusaka (a regional reference laboratory for cysticercosis). The assay involved coating polystyrene 96 well ELISA plates (Nunc® Maxisorp). Two monoclonal antibodies (MoAb) sourced from the Institute of Tropical Medicine (ITM), Antwerp, Belgium were used. The first was MoAb B158C11A10 diluted at 3 *μ*g/mL in carbonate buffer (0.06 M, pH  9.6) and used for coating while the second biotinylated MoAb B60H8A4 diluted at 2.5 *μ*g/mL in Phosphate Buffered Saline-Tween 20 (PBS-T20) + 1% New Born Calf Serum (NBCS) was used as detector antibody. The incubations were carried out at 37°C on a shaker during 30 minutes for the coating of the first MoAb and during 15 minutes for all sub-sequent steps. Blocking was done by adding 150 *μ*L of PBS-T20 + 1% NBCS per well. Without washing the plate, 100 *μ*L of pre-treated sera at a dilution of 1/4 was added to the wells. All steps [addition of second MoAb, streptavidine and Orthophenylene diamine (OPD)] were done after washing the plates five times with PBS-T20. This was followed by addition of 100 *μ*L of biotinylated MoAb B60H8A4 diluted at 3.2 *μ*g/mL in PBS-T20/1% NBCS. Hundred microlitres of streptavidin-horseradish peroxidase (Jackson Immunoresearch Lab, Inc.) diluted at 1/10,000 in PBS-T20/1% NBCS was added to act as conjugate. Thereafter, 100 *μ*L of the OPD solution and H_2_O_2_ was added and incubated without shaking in the dark at room temperature. To stop the reaction, 50 *μ*L of 4N H_2_SO_4_ was added to each well. The plates were read using an ELISA reader at 492 nm. To determine the cut-off, the optical density (OD) of each serum sample was compared with a series of 8 reference negative serum samples (of Ugandan pigs) at a probability level of *P* = .001 [17].

## 3. Results

A total of 513 pigs were sampled in 129 parishes. All parishes in the sample frame were visited. 161 of the samples were collected in the new Kaliro District, with the remainder (352) in Kamuli. For the purposes of this analysis, results for the entire area are considered together. Overall, 74% of pig owners reported that their home had access to a pit latrine, and 18.2% had previously de-wormed their pigs, indicating a relatively low level of veterinary intervention involving the pig population. 

**Figure 2:**
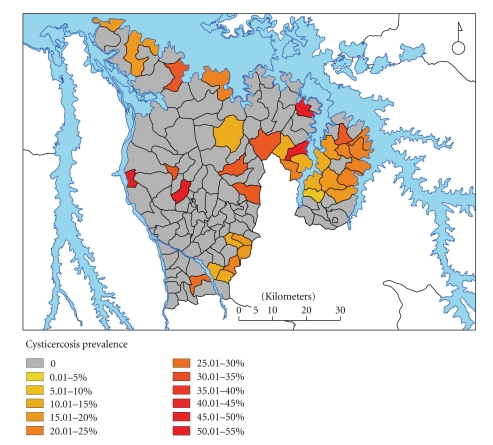
Prevalence of cysticercosis in the study site (Kamuli and Kaliro Districts), by B158/B60 Ag-ELISA.

Of the 513 samples, 480 were screened with the B158/B60 (inadequate volume of serum available in 33 samples). Forty-one pig samples were positive for cysticercosis antigen (see Table 1), or 8.5% (95% CI 6–11%), with no significant differences by age group (*χ* ^2^ = 1.355, *d* *f* = 2, *P* = .508). Positive parishes are shown in Figure 2; 36 of the 129 (28%) parishes in the study area showed at least 1 B158/B60 Ag-ELISA positive pig among the randomly selected individuals sampled from the total population. 

Free range management was found to be the most common method of pig husbandry, based on observations and farmer reports, with the tethering of pigs usually only taking place when neighbours protested against the free roaming of the animals.

## 4. Discussion

Although there are previous studies on porcine cysticercosis in Uganda [18, 19] this is the most recent, albeit preliminary, field survey for porcine cysticercosis in the country, and has investigated the prevalence of antigen-positive pigs across an entire rural district of south-east Uganda. The district under study has seen, in line with many other parts of Uganda, large increases in pig production over the past few years [20]; pigs are relatively cheap and easy to keep in rural areas, where their husbandry has been actively encouraged [3]. The study region is also thought to be a source for pig meat consumed in the capital city, Kampala—pork consumption has been estimated at 5,000 kg per year in pork-roasting centres of Kampala city alone (CW, pers. obs.).

In our field survey, 8.5% of 480 pigs screened were seropositive for the parasite by B158/B60 Ag-ELISA. Preliminary data from other studies conducted in 2008 (FAO TCP/UGA/3104 project; in prep.) indicate similar results by lingual examination, which may indicate a rising prevalence since lingual examination is less sensitive and if the samples from the TCP are subjected to the B158/B60 Ag-ELISA, a higher prevalence might be expected. The results generated in this study showed a lower prevalence than in other studies in ESA in recent years with 23% recorded using Ag-ELISA in Zambia [21]; 17% recorded using lingual examination in Tanzania [6] and 40–50% recorded using Ag-ELISA in Eastern Cape Province, South Africa [22]. These differences are, in themselves, intriguing; latrine provision was reportedly higher in this study than in Zambia, for example, [23], possibly limiting pig access to infectious material. Although in Zambia, latrine provision in itself was not found to be a risk factor for cysticercosis in pigs, the free range method of pig husbandry in the current study implies that those homes not using latrines may be a major risk facilitating access by pigs to infectious material. Cysticercosis infection patterns are known to vary spatially even in fairly restricted areas [11], and infection in pigs is a function of both human contamination of the environment and pig proximity to a human *T. solium * tapeworm carrier [24].

This preliminary study should thus serve as a stimulant for the generation of larger and more comprehensive datasets on this neglected zoonotic disease in Uganda; a particular focus of future work should be on the adult worm and antibody prevalence in humans, as well as risk factors for infection in both pigs and humans. In this regard, further surveys of pigs, seroprevalence surveys in humans and an understanding of cysticercosis-related epilepsy are required, together with risk-factor studies where human infection is found [25]. This will enable a better understanding of the scale of the cysticercosis problem and consequent evidence-based decisions on appropriate methods for its control.

## Figures and Tables

**Table 1:** Prevalence of cysticercosis by B158/B60 Ag-ELISA.

Age categories	No. Screened	No. Seropositive	% seropositive (95%CI)
<3months	99	11	11.1 (4.9–17.3)
>3–12 months	241	17	7.1 (3.8–10.3)
>12 months	140	13	9.3 (4.5–14.1)

Total	480	41	8.5 (6.0–11.0)
